# Tumor Thickness and Histological Grade as Determinants of Sentinel Lymph Node Metastasis in Cutaneous Squamous Cell Carcinoma

**DOI:** 10.3390/medicina62040701

**Published:** 2026-04-06

**Authors:** Irena Janković, Goran Stevanović, Toma Kovačević, Dimitrije Janković, Dimitrije Pavlović

**Affiliations:** 1 Clinic for Plastic and Reconstructive Surgery, University Clinical Center Niš, Bul. Dr Zorana Đinđića 48, 18000 Niš, Serbia; gste66@yahoo.com (G.S.); dimitrije.pavlovic@medfak.ni.ac.rs (D.P.); 2 Department of Surgery and Anesthesiology with Reanimatology, Faculty of Medicine, University of Niš, Bul. Dr Zorana Đinđića 81, 18000 Niš, Serbia; 3 Clinic for Otorhinolaryngology, University Clinical Center Niš, Bul. Dr Zorana Đinđića 48, 18000 Niš, Serbia; tomakovacevic1994@gmail.com; 4 Department of Otorhinolaryngology, Faculty of Medicine, University of Niš, Bul. Dr Zorana Đinđića 81, 18000 Niš, Serbia; 5 Apotekarska Ustanova Niš (Public Pharmacy Institution Niš), Bul. Dr Zorana Đinđića 6, 18000 Niš, Serbia; jankovicdill@gmail.com

**Keywords:** cutaneous squamous cell carcinoma, sentinel lymph node biopsy, tumor thickness, histological grade, lymph node metastasis, risk stratification

## Abstract

*Background and Objectives*: Cutaneous squamous cell carcinoma (cSCC) displays heterogeneous metastatic potential, and the role of sentinel lymph node biopsy (SLNB) in clinically node-negative patients remains debated. To evaluate tumor thickness and histological grade as predictors of sentinel lymph node (SLN) metastasis in high-risk cSCC and to assess the performance of a simplified pathology-based predictive model. *Materials and Methods*: This retrospective single-center study included consecutive patients with high-risk cSCC and clinically N0 status who underwent SLNB. Associations were examined using univariate and multivariable logistic regression, ROC analysis with bootstrap internal validation (2000 iterations), and decision curve analysis. *Results*: Thirty-four patients were analyzed; 12 (35.3%) had SLN metastases. SLN-positive patients had greater tumor thickness (median 5.5 mm vs. 3.0 mm, *p* = 0.006) and higher frequency of G2–G3 histological grade (91.7% vs. 45.5%, *p* = 0.011). Histological grade was the strongest independent predictor in multivariable analysis (OR 14.61, 95% CI 1.63–131.12). The combined model demonstrated apparently high discrimination in this small cohort (AUC 0.91; bootstrap 95% CI 0.79–0.99), though this estimate should be interpreted with caution given the limited number of events. A 4.0-mm threshold yielded sensitivity 83.3% and NPV 86.7%. *Conclusions*: In this exploratory single-center study, tumor thickness and histological grade were complementary predictors of SLN metastasis in cSCC. These findings are preliminary and require validation in larger prospective cohorts.

## 1. Introduction

Cutaneous squamous cell carcinoma (cSCC) of the skin represents a significant proportion of cutaneous malignancies and is associated with considerable morbidity and mortality when metastatic behaviour occurs [1,2]. cSCC accounts for approximately 20–30% of non-melanoma skin cancers in most population-based series, with higher proportions reported in regions with intense ultraviolet exposure [3,4]. Metastatic involvement, particularly of regional lymph nodes, is a well-recognized adverse prognostic factor that substantially increases recurrence risk and disease-specific mortality [5]. Available evidence, including systematic reviews and pooled analyses, has identified several factors associated with lymph node metastasis in cSCC. Among these, tumor thickness and poor histological differentiation have demonstrated independent predictive value in multivariable analyses, while larger tumor diameter and deeper anatomical invasion have been reported as associated features, often correlated with thickness [5,6,7].

Sentinel lymph node biopsy (SLNB) represents a minimally invasive surgical staging procedure originally developed in melanoma and breast cancer to detect occult nodal metastases and guide subsequent management. Although melanoma and cSCC differ substantially in their biological behavior—melanoma is characterized by earlier and more unpredictable hematogenous dissemination, whereas cSCC metastasizes predominantly via regional lymphatics in a more stepwise fashion—this lymphotropic metastatic pattern provides a biological rationale for using SLNB as a staging tool in high-risk cSCC. Nevertheless, unlike in melanoma where SLNB status directly influences systemic treatment decisions, its role in cSCC remains less clearly defined and continues to be debated [8,9]. Early systematic reviews and case series have suggested that SLNB can accurately identify subclinical nodal metastasis in patients with cSCC, with relatively low rates of false negatives and acceptable procedural morbidity when combined with lymphatic mapping techniques [10,11].

Despite these encouraging technical findings, the clinical utility of SLNB in cSCC remains controversial due to heterogeneity in reported outcomes and the absence of large prospective trials [12]. Meta-analyses and pooled series indicate that SLNB positivity rates in high-risk cSCC vary widely across studies, ranging from approximately 8% to 15% in cohorts selected for high-risk features. This wide variation largely reflects differences in patient selection criteria, anatomical site distribution, institutional definitions of high-risk cSCC, and pathological processing protocols—particularly the extent to which serial sectioning and immunohistochemistry are systematically applied for micrometastasis detection [8,10,11]. Some studies have questioned the prognostic value of SLNB, noting that a proportion of patients may develop metastatic disease despite a negative sentinel node, particularly when high-risk tumor characteristics are present [9,10,11,12]. False-negative rates reported in the literature vary across studies and anatomical sites, generally ranging from 5% to 15%; some pooled analyses suggest lower rates in head and neck tumors than in truncal or extremity lesions, likely reflecting differences in lymphatic drainage anatomy and pathological workup protocols [10,11,13].

The lack of consensus is compounded by varying definitions of “high risk” across institutional series and differences in perioperative techniques, patient selection, and follow-up protocols. Recently published systematic reviews emphasize the need to balance the potential staging and prognostic benefits of SLNB against its limitations, including false-negative results and uncertain impact on long-term outcomes such as disease-free or overall survival [13]. Although existing literature generally supports the feasibility and safety of the procedure in selected patients, robust evidence demonstrating that early detection of subclinical nodal metastasis translates into improved clinical outcomes is lacking, underscoring the need for studies that clarify predictors of SLN involvement and refine criteria for SLNB in cutaneous cSCC [13,14].

Among the proposed high-risk features in cSCC, tumor thickness, diameter, histological differentiation, perineural invasion, and immunosuppression have been repeatedly associated with increased metastatic potential [15,16]. Tumor thickness reflects vertical growth and deeper tissue invasion, both of which facilitate access to lymphovascular channels. Histological grade, on the other hand, reflects biological aggressiveness and cellular atypia, potentially indicating greater invasive and metastatic potential [17]. Several institutional cohorts have suggested that poorly differentiated tumors and greater depth of invasion are associated with higher rates of regional nodal metastasis; however, the relative contribution and independent predictive value of these routinely reported parameters remain incompletely clarified, particularly in smaller clinical series [16,17,18].

Tumor thickness and histological grade were selected as the primary variables of interest because both are consistently and reproducibly documented in routine pathology reports across all institutions and require no additional testing or clinical data beyond standard histopathological assessment. Biologically, tumor thickness reflects vertical growth and proximity to lymphovascular channels, facilitating lymphatic dissemination, while histological grade captures intrinsic cellular aggressiveness and invasive capacity. Although other high-risk features—including perineural invasion, lymphovascular invasion, and immunosuppression—are clinically relevant, their documentation is less standardized across institutions, and their inclusion in the primary model was additionally constrained by the limited number of outcome events in the present cohort.

Given that both tumor thickness and histological grade are consistently reported in standard pathological reports, they represent practical, clinically accessible variables for risk stratification [15]. Nevertheless, no widely accepted, clinically simple framework exists that integrates these factors to support decision-making regarding SLNB in cSCC. Additional data from well-characterized cohorts may therefore help refine selection criteria and improve individualized risk assessment [8,11,13].

The aim of this study was to evaluate clinicopathological determinants of sentinel lymph node (SLN) metastasis in patients with cutaneous squamous cell carcinoma. Specifically, we sought to assess the associations among tumor thickness, histological grade, and SLN involvement, determine their independent predictive value, and examine the discriminative performance and clinical utility of a simplified predictive approach based on routinely available pathological parameters.

## 2. Materials and Methods

Study design and setting. This study was conducted at the Clinical Center Niš (Serbia), involving the Clinic for Plastic and Reconstructive Surgery, the Institute of Nuclear Medicine, and the Institute of Pathology. We analyzed consecutive patients with cutaneous squamous cell carcinoma (cSCC) who underwent sentinel lymph node biopsy (SLNB) as part of institutional clinical practice. The study design was retrospective-observational. The study protocol was reviewed and approved by the Ethics Committee of the Faculty of Medicine, University of Niš (protocol number: 01-206-4, approved 18 January 2010), and patient data were anonymized prior to analysis.

Inclusion and exclusion criteria. Eligible participants were patients with histopathologically confirmed high-risk cSCC and clinically N0 disease, with no regional nodal metastases detected on clinical examination or ultrasound. High-risk cSCC was defined by the presence of one or more of the following features: location in the “H-zone” of the face, auricle/periauricular region, scalp, hand, or sun-protected sites; tumor diameter > 2 cm; tumor thickness > 4 mm and/or higher Clark level; ulceration; moderate-to-poor differentiation; immunodeficiency; perineural invasion; or origin in previously damaged skin. Patients with low-risk cSCC, clinically evident nodal metastases, or unavailable sentinel lymph node pathology results were excluded.

In patients with clinical suspicion of cSCC, an excisional biopsy was performed with approximately 5 mm clinical margins into healthy tissue to preserve the quality of lymphatic mapping. After histopathological confirmation of high-risk cSCC and confirmation of N0 status, SLNB was performed. Sentinel lymph node mapping and biopsy. Sentinel node identification was performed using a dual-tracer technique with a radiotracer and blue dye. Lymphoscintigraphy was carried out after intradermal injection of 99mTc-labeled human serum albumin (particle size 200–600 nm), using an activity of 50 MBq in 0.3 mL in a two-day protocol. Dynamic imaging (anterior projection) was performed until visualization of the sentinel node, followed by early static images; late static imaging was performed on the day of surgery, approximately 16–18 h after tracer administration. On the day of surgery, 2 mL of 2% methylene blue was injected intradermally around the biopsy scar approximately 20 min prior to the procedure. SLNB was performed under general anesthesia or local anesthesia with sedation, using a handheld gamma probe for intraoperative detection and excision of the “hot” and/or blue-stained node(s). After node removal, the surgical bed was checked to ensure residual counts were ≤10% of the excised node activity; additional nodes were removed if this criterion was not met. In the same session, the tumor site/scar was excised according to oncologic margins (≥1 cm when feasible).

**Figure 1:**
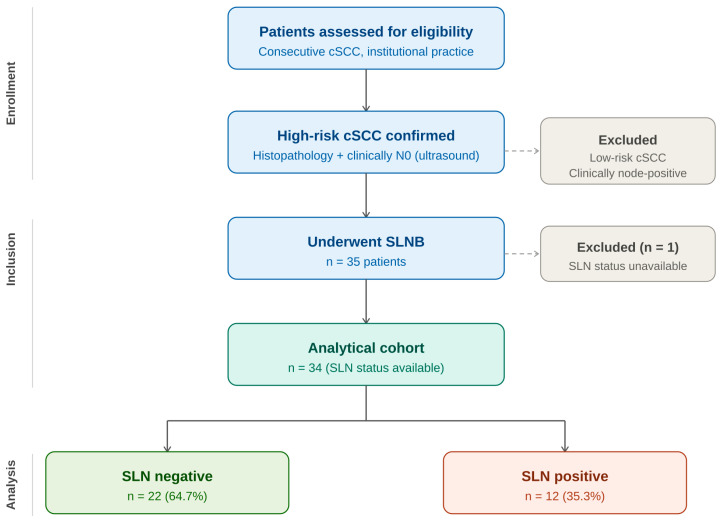
Patient flow diagram.

Excised sentinel lymph nodes and primary tumor specimens were processed as formalin-fixed, paraffin-embedded tissue. Sentinel nodes were evaluated on hematoxylin–eosin (H&E) sections, and immunohistochemistry with pancytokeratin AE1/AE3 was performed when indicated to detect micrometastases. Node sectioning followed a size-adapted protocol (bivalving of nodes < 4 mm; serial sectioning of larger nodes at 2–3 mm intervals). Metastatic deposits were classified as macrometastases (>2 mm) or micrometastases (0.2–2 mm). SLN status was considered positive if metastasis was detected in at least one sampled node. Tumor thickness (in mm) and histological grade were extracted from pathology reports. Tumor thickness was measured from the granular layer of the overlying epidermis—or from the surface of ulceration when present—to the deepest point of invasive tumor, expressed in millimeters, consistent with established dermato-pathological criteria [5]. Grade was coded as [G1–G3] and additionally dichotomized as G2–G3 vs. G1 for descriptive analyses. Histological grade was assessed according to the WHO three-tier classification system: G1 (well-differentiated), G2 (moderately differentiated), and G3 (poorly differentiated), based on the degree of keratinization, nuclear atypia, and mitotic activity [19]. All patients were followed postoperatively as part of routine institutional practice. The median follow-up duration was 14 months (IQR 11–21; range 9–24 months). As this was a retrospective study, pathological assessments were performed by the reporting pathologist at the Institute of Pathology, University Clinical Center Niš, as part of routine clinical practice; formal interobserver agreement assessment was not performed, which represents a limitation of the study. The patient flow through the study is illustrated in Figure 1.

Because multiple lesions may originate from the same patient, analyses were primarily performed at the patient level. For each patient, maximum tumor thickness, highest histological grade, and largest tumor diameter were used to represent the most aggressive tumor characteristics.

Continuous variables were summarized as median (interquartile range) and compared between SLN-positive and SLN-negative groups using the Mann–Whitney U test; categorical variables were compared using Fisher’s exact test. Univariate logistic regression was used to estimate odds ratios (ORs) with 95% confidence intervals (CIs) for each predictor. A multivariable logistic regression model included tumor thickness and histological grade a priori as clinically relevant covariates. Given 12 outcome events, the multivariable model was restricted to two predictors (approximately 6 events per variable) to minimize the risk of overfitting; other clinically relevant variables, including perineural invasion and immunosuppression, could not be incorporated without substantially increasing model instability.

Discrimination was assessed by ROC analysis with AUC estimation; internal validation was performed using bootstrap resampling (2000 iterations) to obtain bias-corrected AUC estimates and 95% CIs. The optimal tumor thickness cut-off was selected using the Youden index, which identifies the threshold that maximizes the sum of sensitivity and specificity (i.e., sensitivity + specificity − 1), providing the best balance between true-positive and false-positive rates. Sensitivity, specificity, PPV, and NPV at this threshold were calculated with 95% CIs using the Wilson method.

Potential clinical utility of the predictive model was assessed using decision curve analysis (DCA). DCA evaluates the net clinical benefit of a predictive model across a range of threshold probabilities—the minimum probability of outcome at which a clinician would intervene—and compares this benefit against two default strategies: treating all patients (universal SLNB) and treating none. A model that consistently outperforms both strategies across a clinically relevant range of thresholds is considered to have potential clinical utility for guiding decisions.

As a secondary analysis, lesion-level data were modeled using generalized estimating equations (GEE) with a logit link and a binomial distribution. GEE was used to account for the correlation among multiple lesions from the same patient by using an exchangeable working correlation structure, thereby providing population-averaged effect estimates robust to intra-patient clustering. All tests were two-sided with *p* < 0.05 considered significant. Statistical analyses were performed using IBM SPSS Statistics (Version 26.0, IBM Corp., Armonk, NY, USA) and Python (Version 3.10) with relevant scientific libraries (pandas, scikit-learn, and statsmodels).

## 3. Results

### 3.1. Patient Characteristics and SLN Status

Thirty-four patients with available SLN status constituted the analytical cohort. One patient was excluded due to unavailable SLN pathology results. Individual clinicopathological characteristics of all 34 patients are presented in Supplementary Table S1. Twelve patients (35.3%) had histologically confirmed SLN metastases. Among SLN-positive cases, metastatic deposits included both micrometastases and macrometastases; a formal subgroup analysis by deposit size category was not performed due to the limited number of events. Individual patient data including tumor site, thickness, histological grade, SLN status, and follow-up are summarized in Supplementary Table S1. Regarding additional high-risk clinicopathologic features, perineural invasion was documented in 1 patient (2.9%) and angiolymphatic invasion in 1 patient (2.9%). Immunosuppression was present in 1 patient (2.9%). Prior tumor recurrence was documented in 2 patients (5.9%). Lymphovascular invasion was not systematically recorded in pathology reports for this retrospective cohort. The high SLN positivity rate observed (35.3%) likely reflects the deliberate enrichment of this cohort for multiple concurrent high-risk features, including high histological grade and greater tumor thickness, rather than the presence of PNI or immunosuppression as dominant drivers. Baseline demographic and tumor characteristics stratified by SLN status are presented in Table 1.

**Figure 2:**
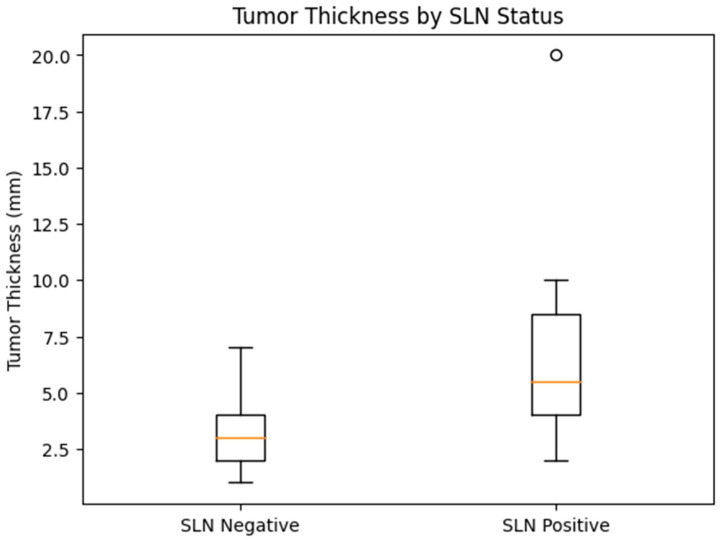
Tumor thickness according to sentinel lymph node (SLN) status (Boxplot representation of maximum tumor thickness (mm) per patient stratified by SLN status. Each box represents the interquartile range (IQR), the central line indicates the median, and whiskers denote the range excluding outliers.

There was no significant difference in age between SLN-positive and SLN-negative patients (median 72 vs. 74 years, *p* = 0.773). Sex distribution was comparable between groups (75.0% vs. 68.2% male, *p* = 1.000). Tumor localization in the head and neck region was frequent in both groups and did not significantly differ (75.0% vs. 63.6%, *p* = 0.705). In contrast, clear differences were observed in the pathological characteristics of the tumors. Patients with positive SLN had significantly greater tumor thickness than those with negative SLN (median 5.5 mm vs. 3.0 mm, *p* = 0.006). Figure 2 illustrates the distribution of maximum tumor thickness per patient stratified by SLN status. Tumor diameter showed a non-significant trend toward higher values in SLN-positive patients (23 mm vs. 19 mm, *p* = 0.143) and was not further pursued in multivariable modeling. Histological grade demonstrated a marked association with SLN involvement. Poorly or moderately differentiated tumors (G2–G3) were substantially more frequent among SLN-positive patients compared to SLN-negative patients (91.7% vs. 45.5%, *p* = 0.011). Overall, tumor thickness and histological grade were the most prominent factors associated with SLN positivity in this cohort, whereas demographic variables and anatomical localization were not significantly associated.

**Figure 3:**
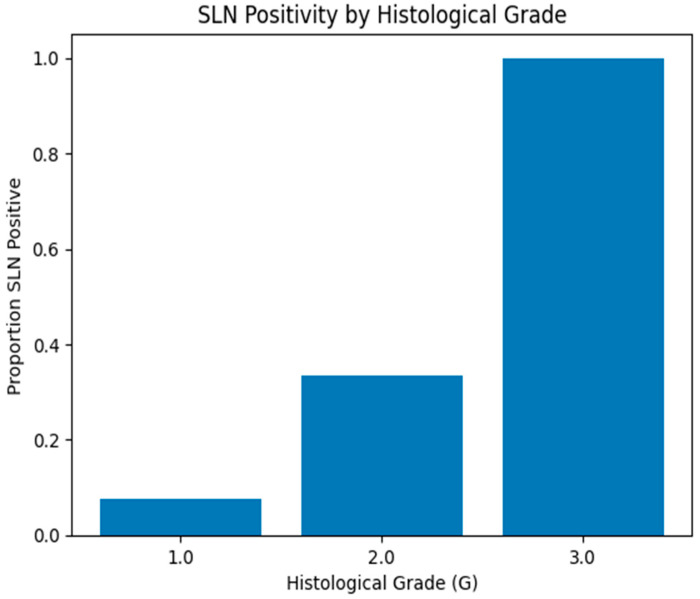
Proportion of SLN positivity according to histological grade. (Bar plot illustrating the proportion of SLN-positive patients across histological grades (G1–G3). A progressive increase in SLN positivity is observed with higher grades of differentiation, with the highest rate in poorly differentiated tumors (G3).

Figure 3 demonstrates the proportion of SLN-positive patients across histological grades. A clear stepwise increase in SLN positivity was observed as grade increased. Among patients with well-differentiated tumors (G1), SLN metastases were infrequent. In contrast, the proportion of SLN positivity increased markedly in moderately differentiated tumors (G2) and was highest in poorly differentiated tumors (G3). When dichotomized, poorly or moderately differentiated tumors (G2–G3) were significantly more common in the SLN-positive group than in the SLN-negative group (91.7% vs. 45.5%, *p* = 0.011).

### 3.2. Univariate Analysis

The results of the univariate logistic regression analysis are presented in Table 2. Tumor thickness was significantly associated with SLN positivity, with each 1 mm increase in tumor thickness corresponding to a 69% increase in the odds of metastatic involvement (OR 1.69, 95% CI 1.11–2.56, *p* = 0.014).

Histological grade demonstrated the strongest individual association with SLN metastasis. Each incremental increase in grade was associated with markedly higher odds of SLN positivity (OR 16.58, 95% CI 2.19–125.34, *p* = 0.006), indicating a substantial impact of tumor differentiation on nodal spread. The wide confidence interval observed for histological grade (95% CI 2.19–125.34) is an expected statistical consequence of the limited number of outcome events (*n* = 12) and should be interpreted with caution. In contrast, demographic variables did not demonstrate meaningful associations. Age was not significantly related to SLN status (OR 0.98 per year, 95% CI 0.91–1.06, *p* = 0.623), and male sex was not associated with increased risk (OR 1.40, 95% CI 0.29–6.83, *p* = 0.677). Tumor localization in the head and neck region also showed no significant relationship with SLN involvement (OR 1.71, 95% CI 0.36–8.23, *p* = 0.501). Overall, tumor thickness and histological grade emerged as the principal factors associated with SLN positivity in univariate analysis, while demographic and anatomical variables did not show statistically significant effects in this cohort.

### 3.3. Multivariable Analysis and Model Discrimination

**Figure 4:**
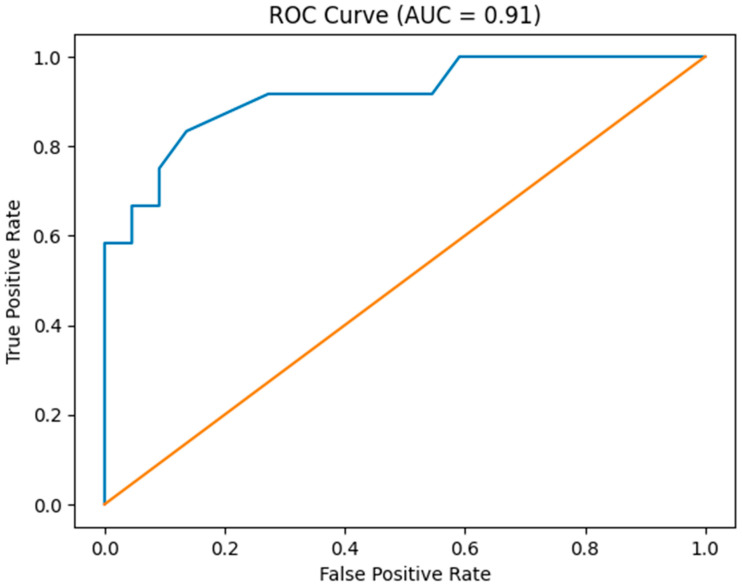
Receiver operating characteristic (ROC) curve of the combined predictive model. The diagonal line represents the reference line of no discrimination.

In the multivariable logistic regression model including tumor thickness and histological grade, histological grade remained an independent predictor of SLN metastasis. Each incremental increase in grade was associated with a more than fourteenfold increase in the odds of SLN positivity (OR 14.61, 95% CI 1.63–131.12, *p* = 0.017). Tumor thickness retained a positive association with SLN involvement; however, it did not reach conventional statistical significance after adjustment for grade (OR 1.61 per 1 mm increase, 95% CI 0.98–2.66, *p* = 0.060). This suggests partial overlap between depth of invasion and histological aggressiveness in explaining metastatic spread. The borderline statistical significance of tumor thickness (*p* = 0.060) should be interpreted cautiously, as it may reflect limited statistical power rather than absence of a clinically meaningful association. The combined model demonstrated apparently high discriminative performance in this small cohort, with an area under the receiver operating characteristic curve (AUC) of 0.91; however, this estimate should be interpreted with caution given the limited number of outcome events. Bootstrap validation yielded a consistent mean AUC estimate (0.91; 95% CI 0.79–0.99); however, the wide confidence intervals reflect substantial uncertainty inherent to the small sample size, and the apparent robustness of discrimination should not be overstated. Overall, histological grade emerged as the most stable independent predictor of SLN metastasis, while tumor thickness provided additional, but partially overlapping, prognostic information. The ROC curve of the combined model is presented in Figure 4.

### 3.4. Diagnostic Performance at the 4.0-mm Threshold

Receiver operating characteristic analysis identified a tumor thickness threshold of 4.0 mm as optimal by the Youden index. At this threshold, sensitivity was 83.3% (95% CI 55.2–95.3), specificity 59.1% (95% CI 38.7–76.7), positive predictive value 52.6% (95% CI 31.7–72.7), and negative predictive value 86.7% (95% CI 62.1–96.3). These findings indicate that a thickness threshold of 4 mm provides strong rule-out capability with a high negative predictive value while maintaining moderate specificity. Although not sufficient as a standalone decision criterion, this cut-off may serve as a clinically practical component of risk stratification when combined with histological grade.

### 3.5. Potential Clinical Utility—Decision Curve Analysis

**Figure 5:**
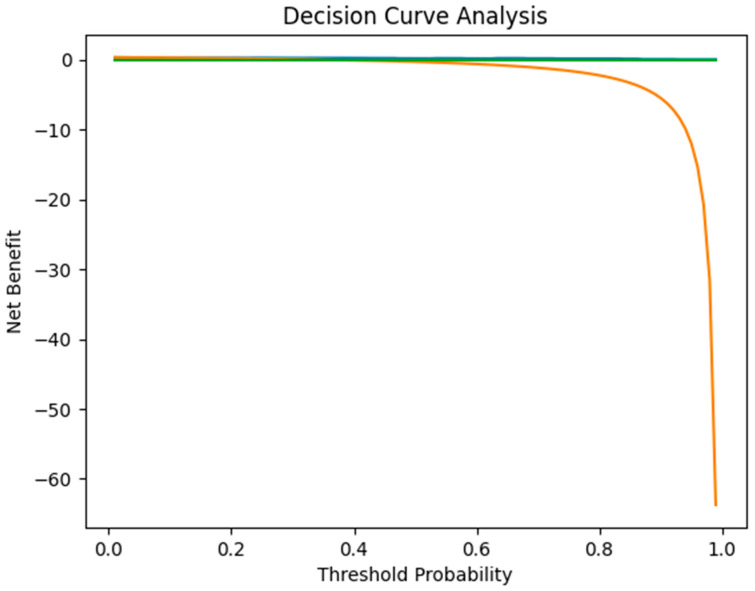
Decision Curve Analysis of the Combined Predictive Model. (Decision curve analysis comparing the multivariable model (tumor thickness + histological grade) with “treat-all” and “treat-none” strategies across a range of threshold probabilities for sentinel lymph node (SLN) metastasis.

Given the small number of outcome events (*n* = 12), the decision curve analysis should be considered exploratory and interpreted with caution; its findings require confirmation in a larger independent cohort. Decision curve (Figure 5) analysis demonstrated that the combined model incorporating tumor thickness and histological grade provided a positive net benefit across a clinically relevant range of threshold probabilities. The model consistently outperformed both the “treat-all” and “treat-none” strategies across approximately 10–60% predicted probability of SLN metastasis. Within this interval, the model improved the balance between identifying true-positive cases and avoiding unnecessary procedures, indicating potential clinical utility for guiding decisions regarding SLN biopsy. At lower thresholds, the model reduced overtreatment compared with a universal biopsy approach, while at higher thresholds, it remained superior to a no-biopsy strategy. These findings are hypothesis-generating only and should not be interpreted as evidence of established clinical applicability; confirmation in larger, prospective, independent cohorts is required before any clinical translation can be considered.

### 3.6. Secondary GEE Analysis

Our secondary lesion-level analysis using generalized estimating equations supported the robustness of the thickness association after accounting for intra-patient clustering. Although histological grade was no longer statistically significant in this clustered framework, the effect size remained large and directionally consistent, with wide confidence intervals that likely reflect limited power and within-patient correlation rather than an absence of biological association. This result is methodologically plausible in datasets where grade varies little within patients and events are few, and it reinforces that the principal signals in our data are driven by thickness and aggressive histology.

## 4. Discussion

Given the exploratory nature of this single-center study and the limited sample size, all findings should be considered preliminary and hypothesis-generating rather than definitive. The key findings of this exploratory study are threefold: (1) tumor thickness and histological grade were the only variables significantly associated with SLN positivity; (2) histological grade was the strongest independent predictor in multivariable analysis; and (3) the combined model demonstrated apparently high discriminative performance in this small cohort (AUC 0.91), with a preliminary signal of potential clinical utility as suggested by the exploratory decision curve analysis. In this exploratory single-center cohort of cSCC, tumor thickness and histological grade emerged as the most informative routinely available predictors of sentinel lymph node (SLN) metastasis. SLN-positive patients presented with significantly greater tumor thickness, and a higher histological grade was markedly enriched among metastatic cases. In multivariable modeling, histological grade retained an independent association with SLN involvement, whereas thickness remained positively associated but attenuated after adjustment, suggesting partial overlap between depth of invasion and intrinsic tumor aggressiveness in explaining nodal spread. These findings are clinically appealing because both thickness and grade are consistently available in standard pathology reports and require no additional testing.

From a biological perspective, greater tumor thickness indicates deeper invasion into the dermis and subcutis, where lymphatic vessel density is substantially higher, thereby facilitating access to lymphatic channels and regional nodal spread. Histological grade captures the degree of tumor dedifferentiation, which is mechanistically linked to enhanced metastatic capacity. Poorly differentiated cSCC exhibits reduced E-cadherin expression and increased vimentin co-expression, consistent with partial epithelial–mesenchymal transition—a process associated with loss of intercellular adhesion, increased invasiveness, and lymphovascular permeation [20,21,22]. These molecular changes collectively facilitate the stepwise dissemination of poorly differentiated tumor cells to regional lymph nodes.

Our results align with the broader literature, which emphasizes that only a subset of cSCCs behaves aggressively, yet outcomes are poor once regional or distant metastasis occurs. For reference, Allen and Stolle reported a pooled sensitivity of 79% and negative predictive value of 96% for SLNB across published cSCC series [15], while Lhote et al. observed a SLN positivity rate of 14% in a comparable high-risk cohort [23]—substantially lower than the 35.3% observed in our enriched series. To our knowledge, no published study has reported a combined predictive model AUC for tumor thickness and histological grade as joint predictors of SLN positivity in cSCC, precluding direct numerical AUC comparison; our finding of an AUC of 0.91 therefore represents a novel contribution, though one requiring prospective validation. These findings are consistent with the established literature that identifies tumor thickness and poor differentiation as key determinants of regional metastasis in cSCC [24,25,26]. Importantly, our diagnostic performance analysis demonstrated that this threshold favored rule-out, with a high negative predictive value and acceptable sensitivity. This suggests that tumor thickness may function as a pragmatic screening element in clinical decision-making, particularly when integrated with histological grade. Thickness alone is unlikely to suffice as an indication for SLNB, but in combination with poor or moderate differentiation, it provides a clinically interpretable and reproducible framework for risk stratification [12]. Within this context, our finding that thickness was associated with SLN positivity, together with the independent signal of histological grade, is consistent with a biological model in which deeper invasion facilitates lymphatic access while poor differentiation reflects intrinsic invasive capacity.

Risk stratification in cSCC has evolved toward staging frameworks that incorporate multiple adverse pathological features. The Brigham and Women’s Hospital (BWH) staging system was specifically developed to improve prognostic discrimination compared with the AJCC/UICC system [27]. The BWH system defines four high-risk factors: tumor diameter ≥ 2 cm, poor histological differentiation, perineural invasion of named nerves (diameter ≥ 0.1 mm), and invasion beyond subcutaneous fat. Tumors are staged as T1 (zero high-risk factors), T2a (one high-risk factor), T2b (two or three high-risk factors), and T3 (four high-risk factors or bone invasion). Studies evaluating nodal staging in BWH T2b and T3 tumors have reported markedly higher rates of occult nodal disease—ranging from 20–30%—compared with T1 and T2a tumors, supporting the rationale for selective SLNB in carefully defined high-risk subsets [27,28].

The simplified two-variable model proposed in the present study—based on tumor thickness and histological grade—captures two of the four BWH high-risk factors using only routine pathological data, without requiring clinical information on perineural invasion or anatomical depth of invasion beyond subcutaneous fat. While the BWH system provides a more comprehensive multi-factor staging framework, its application requires complete documentation of all four risk factors, which may not always be systematically available in retrospective datasets or smaller institutions. Our approach offers a complementary, pathology-driven risk stratification tool that can be applied when complete BWH staging data are unavailable, though it should not be regarded as a replacement for comprehensive risk assessment. Our cohort, enriched for aggressive pathological features, likely corresponds biologically to the BWH T2b–T3 categories, which may partly explain the elevated SLN positivity rate observed compared with pooled estimates in unselected populations. This is relevant because the current clinical dilemma is not whether nodal metastasis matters—it clearly does—but rather which clinically node-negative patients warrant nodal staging and intensified surveillance. Recent analyses emphasize that a nontrivial proportion of adverse outcomes can occur even at lower stages, reinforcing the need for pragmatic selection strategies that remain interpretable and reproducible in routine practice [28,29].

The role of SLNB in cSCC remains controversial. While SLNB is technically feasible with high identification rates in modern series and meta-analyses, reported SLN positivity varies substantially across studies, reflecting heterogeneity in patient selection, anatomic sites (including head and neck), and definitions of “high risk” [8]. In single-center cohorts enriched for high-risk features, positivity rates often fall in the low-to-mid teens (e.g., ~11–15%), though they may increase when more intensive pathologic processing and immunohistochemistry are systematically applied [30]. Conversely, other data suggest substantially higher positivity in very-high-risk subsets defined by staging systems (e.g., reports describing markedly higher positivity in BWH T2b tumors), again underscoring that selection criteria largely drive observed yields [31]. Our SLN positivity rate of 35.3% substantially exceeded pooled estimates from unselected high-risk series (~8–15%). Three factors likely contribute to this discrepancy: first, our cohort was deliberately enriched for very-high-risk clinicopathological features, biologically corresponding to BWH T2b–T3 categories where positivity rates of 20–30% have been reported; second, the small sample size renders the observed proportion susceptible to selection bias and wide sampling variability; and third, the use of immunohistochemistry when indicated for micrometastasis detection may have increased the detection yield compared with series relying exclusively on hematoxylin–eosin evaluation.

Beyond yield, another key reason for debate is the uncertainty regarding the downstream outcome impact. In some analyses combining institutional series with published cases, SLN status did not clearly translate into differences in relapse-free or overall survival, which has been interpreted as insufficient evidence to support routine SLNB in SCC [23]. Furthermore, observational comparisons in selected high-thickness cSCC cohorts have not consistently shown lower rates of metastatic progression in SLNB-managed patients than in observation, raising questions about whether earlier detection of occult nodal disease necessarily improves long-term outcomes in this setting [28]. Taken together, existing evidence supports SLNB as a feasible staging tool but leaves uncertainty about whether it should be routinely applied outside carefully selected high-risk populations.

A further practical limitation is the possibility of false-negative or “false-omission” results, which can occur due to technique, the complexity of lymphatic drainage (particularly in the head and neck), and pathological assessment. Detailed institutional work has shown that more thorough tissue processing and immunohistochemistry can reclassify initially negative sentinel nodes, increasing observed positivity and highlighting the sensitivity of reported yields to pathology protocols [30]. Clinically, this matters because a negative SLNB does not fully eliminate the risk of subsequent metastasis in high-risk disease; prior series have reported nodal progression despite negative SLNB in tumors with risk factors such as thickness > 4 mm or recurrence, underscoring that surveillance and clinical judgment remain essential even after negative staging [24,32,33].

In this context, our identification of a 4.0-mm tumor thickness threshold is clinically intuitive and concordant with widely cited high-risk definitions that use >4 mm depth/thickness as a marker of increased metastatic risk [24,25]. Importantly, in our cohort, the diagnostic profile at this threshold favored rule-out performance (high NPV with moderate specificity), supporting the concept that thickness may serve as a pragmatic screening component rather than a standalone indication for SLNB. This is aligned with the broader message in high-risk cSCC reviews: millimeter thickness thresholds are useful for identifying higher-risk strata but require integration with additional adverse features–such as poor differentiation, perineural invasion, or immunosuppression–to guide management decisions [34]. Our results specifically underscore the complementary role of histological grade, which remained strongly associated with SLN metastasis in the multivariable model.

Although our study does not include long-term survival outcome data, the published literature consistently demonstrates that both tumor thickness and histological grade are independently associated with adverse oncologic outcomes in cSCC. Brantsch et al. reported in a large prospective cohort that tumor thickness greater than 2 mm was significantly associated with higher rates of recurrence and disease-specific death, with risk increasing progressively with greater depth of invasion [5]. Poor histological differentiation has similarly been identified as an independent predictor of disease-specific mortality and regional metastasis across multiple large institutional cohorts and meta-analyses [17,18]. In our own cohort, the single disease-related death occurred in the patient with macrometastatic SLN involvement—consistent with the known adverse prognostic impact of nodal metastasis in cSCC. Taken together, these data provide biological and prognostic support for the predictive associations identified in the present study, and underscore the clinical relevance of tumor thickness and histological grade as early indicators of metastatic potential.

Beyond association testing, we evaluated both discrimination and potential clinical utility. The combined thickness-plus-grade model demonstrated apparently favorable discrimination in this small cohort, though the wide bootstrap confidence intervals reflect the inherent uncertainty of AUC estimation with limited events. Decision curve analysis provided a preliminary signal of potential net clinical benefit across plausible threshold probabilities, but this finding is exploratory and its clinical significance remains uncertain pending external validation. This approach is particularly relevant given the variability in SLNB yield and the lack of consensus on selection criteria: rather than advocating indiscriminate SLNB, our findings support a pragmatic, pathology-driven risk-assessment strategy based on parameters already available in routine care.

Several limitations should be acknowledged. The cohort is small, resulting in wide confidence intervals and increased susceptibility to selection bias, particularly with respect to indications for SLNB. The retrospective, single-center design and the absence of external validation limit generalizability and preclude claims of transferable predictive performance. In addition, we did not include other established high-risk features, such as perineural invasion, lymphovascular invasion, or immunosuppression, in the primary multivariable model, as event counts limited model complexity. A further methodological limitation is the reliance on a single immunohistochemical marker (CK AE1/AE3) for the detection of micrometastases. While CKAE1/AE3 is widely used, it may produce false-positive results due to non-specific staining of interstitial reticulum cells in lymph nodes. The use of a complementary squamous-specific marker (e.g., p40, p63, or CK5/6) would have been preferable to increase specificity. This represents an institutional protocol limitation applicable to the study period. Immunohistochemistry for micrometastasis detection was performed when indicated by the reporting pathologist rather than according to a pre-specified systematic protocol, which may have resulted in underdetection of micrometastases in a subset of cases. Sentinel lymph nodes were processed using size-adapted sectioning at 2–3 mm intervals, which may have resulted in the omission of micrometastases smaller than the sectioning interval. A more intensive serial sectioning protocol would have been preferable for maximizing micrometastasis detection sensitivity. Due to the limited number of SLN-positive cases (*n* = 12), a formal comparative analysis between micrometastases and macrometastases was not statistically feasible; results should be interpreted accordingly. Follow-up data were available for all 34 patients, with a median duration of 14 months (IQR 11–21; range 9–24 months). During this period, one patient died (the single case with macrometastasis), and no regional nodal recurrence was documented among SLN-negative patients. However, the relatively short follow-up duration precludes definitive assessment of long-term oncologic outcomes, including disease-free and overall survival, and results should be interpreted accordingly. The small number of SLN-positive cases (*n* = 12) leads to imprecision in estimating the odds ratio, as reflected by the wide confidence intervals in both univariate and multivariable analyses. Similarly, the borderline significance of tumor thickness in the multivariable model (*p* = 0.060) likely reflects insufficient statistical power due to the small sample size rather than a true absence of effect. Decision curve analysis was performed on only 12 outcome events, which may render the net benefit estimates unreliable; these results should be considered exploratory and require validation in larger cohorts. Nevertheless, by focusing on two widely available pathological variables, providing internal validation, quantifying threshold-based diagnostic performance, and exploring potential clinical utility through decision curve analysis, this study offers a clinically interpretable framework that complements existing high-risk definitions and highlights the potential value of integrating thickness and grade in SLNB decision-making.

## 5. Conclusions and Future Directions

In this exploratory single-center study, tumor thickness and histological grade emerged as complementary, routinely available predictors of sentinel lymph node metastasis in high-risk cutaneous squamous cell carcinoma. Histological grade was the strongest independent predictor in multivariable analysis, whereas the 4.0-mm tumor thickness threshold showed a favorable rule-out profile with a high negative predictive value. The combined model demonstrated apparently high discrimination in this small exploratory cohort (AUC 0.91), with a preliminary signal of potential clinical utility across plausible decision thresholds as suggested by the exploratory decision curve analysis; these findings require confirmation in larger prospective studies. These findings support a pragmatic, pathology-driven approach to patient selection for SLNB in clinically node-negative patients, focused on parameters that are universally and reproducibly available in routine practice.

Given the small sample size, retrospective single-center design, and absence of external validation, all findings should be regarded as preliminary and hypothesis-generating rather than definitive. Overstated conclusions are not warranted at this stage, and cautious interpretation is essential.

Future research should prioritize: (1) prospective multicenter validation of the proposed thickness threshold and the independent predictive role of histological grade in larger, well-characterized cohorts; (2) development of more comprehensive multivariable models incorporating additional established high-risk features—including perineural invasion, lymphovascular invasion, and immunosuppression status—once sufficient event counts permit; and (3) systematic evaluation of long-term oncologic outcomes, including disease-free survival, overall survival, and nodal recurrence rates, in relation to SLN status and primary tumor pathological characteristics.

## Figures and Tables

**Table 1:** Baseline Characteristics of Patients According to Sentinel Lymph Node (SLN) Status.

Variable	SLN Negative (*n* = 22)	SLN Positive (*n* = 12)	*p*-Value
Age (years), median (IQR)	74 (64–76)	72 (63–75)	0.773
Tumor thickness (mm), median (IQR)	3.0 (2.0–4.0)	5.5 (4.0–8.5)	0.006
Tumor diameter (mm), median (IQR)	19 (11–22)	23 (17–30)	0.143
Male sex, *n* (%)	15 (68.2%)	9 (75.0%)	1
Head and neck location, *n* (%)	14 (63.6%)	9 (75.0%)	0.705
Histological grade G2–G3, *n* (%)	10 (45.5%)	11 (91.7%)	0.011

**Table 2:** Univariate Logistic Regression Analysis for Prediction of SLN Positivity.

Predictor	Odds Ratio (OR)	95% Confidence Interval	*p*-Value
Tumor thickness (per 1 mm)	1.69	1.11–2.56	0.014
Tumor diameter (per 1 mm)	1.07	0.99–1.16	0.1
Histological grade (per 1 grade increase)	16.58	2.19–125.34	0.006
Age (per 1 year)	0.98	0.91–1.06	0.623
Male sex (vs. female)	1.4	0.29–6.83	0.677
Head and neck location (vs. other sites)	1.71	0.36–8.23	0.501

## Data Availability

The data that support the findings of this study are available on request from the corresponding author.

## Supplementary Materials

The following supporting information can be downloaded at: https://www.mdpi.com/article/10.3390/medicina62040701/s1, Table S1: Individual clinicopathological characteristics of all 34 patients in the analytical cohort.
