# Supplementary material for: Tumor Thickness and Histological Grade as Determinants of Sentinel Lymph Node Metastasis in Cutaneous Squamous Cell Carcinoma
Source: Medicina (Kaunas). 2026 Apr 6;62(4):701. doi: 10.3390/medicina62040701 (PMC13117494; doi:10.3390/medicina62040701)
Supplement: Supplementary file 1 [file medicina-62-00701-s001.zip › medicina-4198168-supplementary.pdf]

Supplementary Table S1. Individual clinicopathological characteristics of all 34 patients in the analytical cohort.

Pt.	Age (yrs)	Sex	Tumor site	Diameter (mm)	Thickness (mm)	Grade	PNI	Immuno-suppression	SLN status	Metastasis type	DFS (mo)	Deceased
1	55	M	Back	7	4	G2	No	No	Negative	—	24	No
2	68	M	Ear	20	3	G2	No	No	Negative	—	24	No
3	53	M	Forearm	17	2	G2	No	No	Negative	—	24	No
4	71	F	Lateral cheek	22	3	G1	No	No	Negative	—	23	No
5	76	M	Posterior scalp	35	4	G2	No	No	Positive	Micro	23	No
6	74	M	Hand	20	6	G1	No	No	Negative	—	22	No
7	75	F	Hand	17	6	G2	No	No	Negative	—	21	No
8	82	M	Lateral cheek	17	7	G2	No	No	Negative	—	21	No
9	78	F	Forehead	20	5	G1	No	No	Negative	—	20	No
10	73	M	Anterior scalp	20	3	G1	No	No	Negative	—	20	No
11	82	M	Forehead	21	5	G2	No	No	Positive	Micro	21	No
12	84	F	Neck	35	5	G2	Yes	No	Negative	—	20	No
13	68	M	Chest wall	22	2	G1	No	No	Negative	—	19	No
14	80	M	Lateral cheek	25	8	G2	No	No	Positive	Sub-capsular	19	No
15	48	M	Foot	20	10	G2	No	Yes	Positive	Sub-capsular	16	No
16	72	M	Hand	25	2	G2	No	No	Negative	—	17	No
17	63	M	Back	20	1	G1	No	No	Negative	—	16	No
18	58	F	Temple	8	4	G1	No	No	Positive	Micro	12	No
19	74	F	Forehead	35	2	G1	No	No	Negative	—	12	No
20	63	M	Ear	30	8	G3	No	No	Positive	Sub-capsular	12	No
21	78	M	Retroauricular	18	2	G1	No	No	Negative	—	12	No
22	75	M	Chest wall	65	20	G3	Yes*	No	Positive	Macro	9	Yes
23	76	M	Temple	10	4	G1	No	No	Negative	—	11	No
24	65	F	Nose	30	2	G3	No	No	Positive	Sub-capsular	11	No

Pt.	Age (yrs)	Sex	Tumor site	Diameter (mm)	Thickness (mm)	Grade	PNI	Immuno-suppression	SLN status	Metastasis type	DFS (mo)	Deceased
25	60	F	Medial cheek	15	3	G1	No	No	Negative	—	12	No
26	60	F	Periorbital	8	1	G1	No	No	Negative	—	11	No
27	76	M	Upper arm	23	4	G2	No	No	Negative	—	11	No
28	80	M	Posterior scalp	10	3	G2	No	No	Negative	—	10	No
29	63	M	Back	32	4	G3	No	No	Positive	Micro	10	No
30	75	M	Neck	8	2	G1	No	No	Negative	—	9	No
31	69	M	Ear	15	3	G3	No	No	Positive	Micro	10	No
32	58	M	Medial cheek	10	4	G2	No	No	Negative	—	9	No
33	74	F	Chin	18	10	G2	No	No	Positive	Sub-capsular	9	No
34	75	M	Ear	8	6	G3	No	No	Positive	Sub-capsular	9	No

Pt. = patient number; PNI = perineural invasion; SLN = sentinel lymph node; DFS = disease-free survival; mo = months; Micro = micrometastasis (0.2–2 mm); Macro = macrometastasis (>2 mm); Sub-capsular = sub-capsular deposit; — = not applicable (SLN negative).

** Angiolymphatic invasion present. Yes* = immunosuppression due to prior organ transplant.*
